# DNA Comet Giemsa Staining for Conventional Bright-Field Microscopy

**DOI:** 10.3390/ijms15046086

**Published:** 2014-04-10

**Authors:** Andreyan Osipov, Ekaterina Arkhangelskaya, Alexei Vinokurov, Nadezhda Smetanina, Alex Zhavoronkov, Dmitry Klokov

**Affiliations:** 1 State Research Center—Burnasyan Federal Medical Biophysical Center of Federal Medical Biological Agency (SRC-FMBC), 46, Zhivopisnaya Str., Moscow 123098, Russia; E-Mails: ek.arkhangelskaya@gmail.com (E.A.); smetaninanm@gmail.com (N.S.); 2 Dima Rogachev Federal Research Center of Pediatric Hematology, Oncology and Immunology, 1, Samora Machel Str., Moscow 117997, Russia; E-Mails: a_vinokurov@inbox.ru (A.V.); alex@biogerontology.org (A.Z.); 3 Chalk River Laboratories, Atomic Energy of Canada Limited, Stn 51 Plant Road, Chalk River, ON K0J1P0, Canada; E-Mail: klokovd@aecl.ca

**Keywords:** Giemsa stain, comet assay, bright-field microscopy, DNA damage

## Abstract

This study was undertaken to evaluate the compatibility of Giemsa staining protocol with the comet assay. We showed, for the first time, that DNA comets can be visualized and analyzed using Giemsa staining. We generated DNA damage dose response curves for human peripheral blood lymphocytes exposed to X-ray radiation using the comet assay with either SybrGreen I or Giemsa stain. The dose response curves were fitted by linear regressions (*R*^2^ > 0.977). The SybrGreen I results showed only ~1.2-fold higher slope coefficient (method sensitivity) compared to the Giemsa results. The unexpectedly high sensitivity of Giemsa staining for the comet assay is due to the Romanowsky-Giemsa effect, the stain photo-stability and the higher resolution of bright-field imaging compared to fluorescence imaging. Our results demonstrate that Giemsa staining can effectively be used for measuring DNA damage by the comet assay. The low cost and availability of Giemsa stain makes this method affordable for any low budget research and will facilitate new applications of the comet assay in biology and medicine.

## 1. Introduction

Measuring DNA damage is a key step in a broad range of biomedical research studies. Among several methods of detecting DNA damage, the comet assay, being very simple, cheap and not requiring sophisticated high-cost equipment, has been most widely adapted and used. This method was first described by Ostling and Johanson in 1984 as a single cell gel electrophoresis method [1] and involved embedding cells into agarose gels, followed by lysis and electrophoresis. Stained with acridine orange, the resulting cells resembled comets giving rise to the term “comet assay” and that has become the common name for the assay. Indeed, the intact high-molecular weight genomic DNA does not migrate in the agarose gel electrophoresis forming the “head” of a comet, whereas damage-relaxed DNA loops and DNA fragments migrate away from the head forming the “tail” of the comet. The originally described method consisted of neutral pH lysis and electrophoresis. In 1988 Singh *et al*. developed an alkaline version of the comet assay carried out at pH > 13, allowing for detection of DNA single strand breaks and alkali-labile sites [2]. The alkaline version of the assay allowed detecting DNA damage produced by as low as 25 cGy. Currently, there are numerous comet assay modifications developed for the detection of very specific DNA lesions and their repair rates, such as single- and double-strand breaks, various base lesions, DNA cross-links, DNA-protein cross-links, *etc*., in many cell types [3–9]. To achieve high method sensitivity, high quality visualization and imaging of comets is required. This represents a limitation to its widespread use since many laboratories are not equipped with fluorescence microscopes and high-sensitivity CCD cameras for capturing fluorescent images of DNA comets. The only known method of staining and visualizing DNA comets for bright-field microscopy is a modified silver staining method [10–12]. However, the modified silver staining protocol consists of multiple sensitive steps prone to variation and is not easy to conduct in small laboratories which do not have the money to purchase relatively expensive commercial kits.

In this report we show, for the first time, that DNA comets can effectively be stained with an inexpensive and versatile Giemsa stain (azur-eosin-methylene blue solution) widely used in many cyto- and histochemical applications. The sensitivity of Giemsa staining for the comet assay was sufficient not only for the visualization of the comets, but also for imaging and analysis by specific comet assay software.

## 2. Results and Discussion

In 2003, Fernandez *et al*. reported the use of a commercially available version of the Romanowsky stain, Diff-Quick, for DNA staining in the sperm chromatin dispersion test [13]. A year later, they used Wright staining for the same purpose [14]. Both staining methods allowed visualization of freely moving DNA loops around sperm cells. We were intrigued as to whether Giemsa staining, which is similar to the Diff-Quick and Wright stains, could be used for DNA staining in the comet assay.

As an object of experimentation in this study, human peripheral blood lymphocytes were chosen. These cells represent a convenient model and are often used in biomedical research involving the comet assay [15].

**Figure 1.**
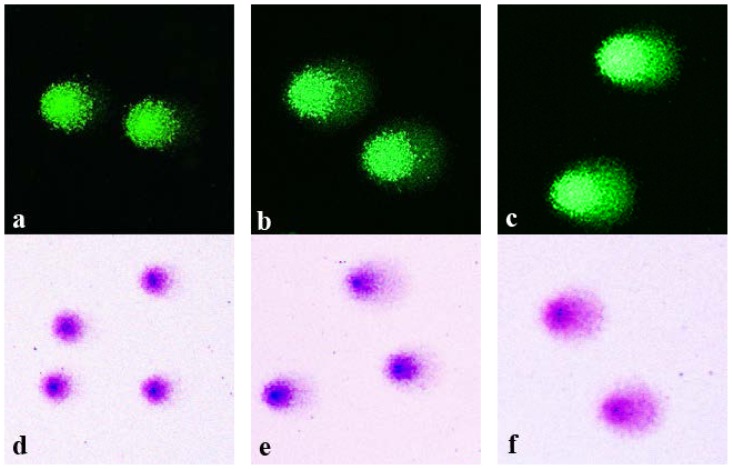
Representative images of DNA comets, obtained from human peripheral blood lymphocytes, stained with SybrGreen I (**a**–**c**) and Giemsa (**d**–**f**). (**a**) and (**d**), control non-irradiated cells; (**b**) and (**e**), cells irradiated with 3 Gy of X-rays; (**c**) and (**f**), cells irradiated with 6 Gy of X-rays.

Isolated and agarose-embedded lymphocytes were exposed to X-ray irradiation with 1–6 Gy doses. Following the standard comet assay procedures (cell lysis, DNA unwinding, electrophoresis and neutralization), the comets were fixed in 70% ethanol. Replicate slides were stained in parallel with either highly sensitive fluorescent dye SybrGreen I or conventional Giemsa stain. Shown in Figure 1 are representative microphotographs of the comets generated from the control non-irradiated cells (Figure 1a,d) and cells irradiated with either 3 Gy (Figure 1b,e) or 6 Gy (Figure 1c,f). DNA comets stained with SyrbGreen I are shown in panels Figure 1a–c, whereas Giemsa-stained comets are shown in panels Figure 1d–f.

As expected, irradiation of the cells produced dose-dependent increases in the fraction of DNA that was electrophoretically-mobile. It is evident from Figure 1 that the appearance of the comets visualized by SybrGreen I and by Giemsa are similar. DNA in the comet tails stained with Giemsa was readily visualized. It is noteworthy that the color of Giemsa-stained comets varied from dark blue in centers of the comet heads to pink-purple in the tails. This color variation is related to the concentration of DNA and its conformation. Interestingly, the coloring of Giemsa-stained comets was reminiscent of color patterns typically observed in the G-banding assay, the technique of Giemsa staining of condensed chromosomes following a treatment with trypsin or alkali, resulting in different coloration of the active euchromatin *vs*. inactive heterochromatin [16]. It seems possible that Wright-Giemsa staining may provide additional information about the structure of DNA in various components of the DNA comets.

**Figure 2.**
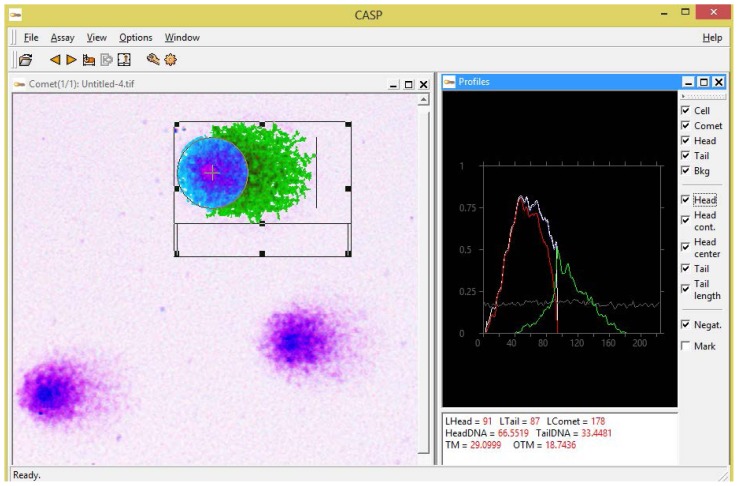
A snapshot of CASP 1.2.2 software (CASPlab, Wroclaw, Poland) showing a Giemsa-stained DNA comet processed by the software. In the lower right corner, various parameters of the comet highlighted in the center window of the software, such as Olive tail moment, tail moment, % DNA in tail, *etc*., are displayed.

Next, to quantify the amount of DNA damage we analyzed the comets obtained with SybrGreen I *vs*. Giemsa staining using CASP 1.2.2 software (CASPlab, Wroclaw, Poland). The choice of this particular comet assay software was mostly dictated by the fact that it is freely available for download on the Internet (http://casplab.com), which is yet another factor allowing positioning of our modification of the comet assay as an affordable alternative for low-budget laboratories. A software snapshot shown in Figure 2 demonstrates an example of the analysis of a Giemsa-stained comet using CASP software.

**Figure 3.**
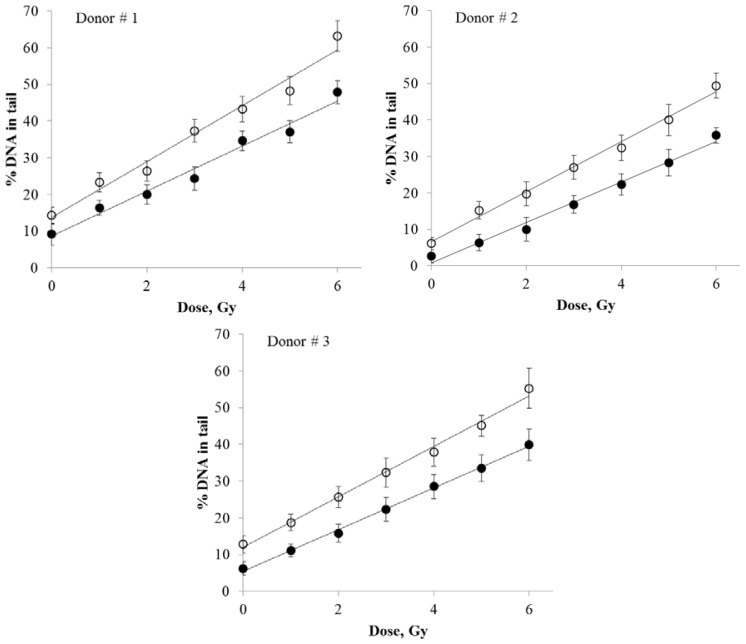
The dose response curves for DNA damage in X-ray irradiated human peripheral blood lymphocytes from three donors measured by quantifying the percentage of DNA in comet tails. DNA comets prepared as described in Materials and Methods were stained with either SybrGreen I (open circles, curve **1**) or Giemsa (closed circles, curve **2**). The data were fitted with linear regressions. Means of three measurements (three slides per treatment) ± standard errors are shown.

DNA damage produced in human peripheral blood lymphocytes by various doses of X-rays was quantified using either SybrGreen I or Giemsa stain. The resulting dose response curves are presented in Figure 3. The regression analysis of the data resulted in linear fits for both staining protocols (*R*^2^ > 0.977).

The detailed results of the regression analysis are presented in Table 1. The slope coefficients (*b*) in linear regression equations (*y* = *a* + *bx*, where “*y*” is the percentage of DNA in the comet tails and “*x*” is dose in Gy) define the rate of increase in DNA damage as a function of dose. The SybrGreen I results showed ~1.2-fold higher slope coefficient compared to the Giemsa results (Donor #1: *b*(SybrGreen I)/*b*(Giemsa) = 1.24; Donor #2: 1.23; Donor #3: 1.21), indicating that Giemsa staining was ~1.2-fold less sensitive than the SybrGreen I protocol. The differences in the dose response slopes between the two staining protocols were statistically significant (Z-test, Table 1). Overall, these data suggest that Giemsa staining is an acceptable alternative to fluorescence staining. The unexpectedly high sensitivity of Giemsa stain for the comet assay, as demonstrated in our experiments, may be related to its photostabilty (the lack of photobleaching) and higher resolution and sensitivity of an image capturing system for bright-field microscopy compared to fluorescence microscopy.

**Figure 4.**
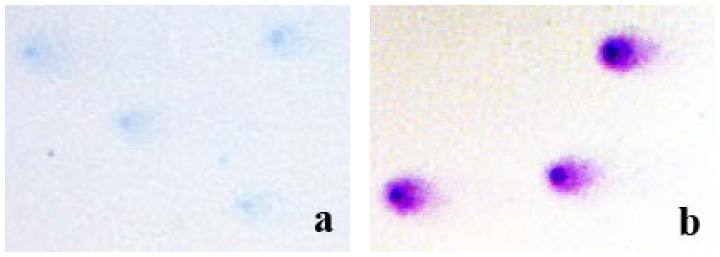
Representative microphotographs of DNA comets obtained from human peripheral blood lymphocytes exposed to 4 Gy of X-rays and stained with either azure B (**a**) or Giemsa (**b**). Both pictures were taken at the same magnification (×200).

The main components of the Wright-Giemsa stain are azure B (CI52010) and eosin Y (CI45380). Given the fact that only azure B directly binds to DNA, one would expect that Giemsa staining should result in blue colored DNA. However, due to the effect of Romanowsky-Giemsa the observed color of Giemsa-stained DNA and nuclei is bright purple. Interestingly, the chemical nature of this effect has been a subject of studies for over 100 years [17,18]. The purple color of Giemsa-stained DNA is caused by eosin Y that has an absorption maximum at 552 nm (the so called Romanowsky band). Formation of DNA-azure B-eosin Y complexes was shown in solutions [17]. The complexes consisted of two eosin Y molecules and one azure B molecule intercalated into the double helix structure of DNA; however the azure B-eosin Y complexes catalyzed by DNA could dissociate from DNA leaving space for another round of azure B-eosin Y complex formation. Therefore, a chain reaction can be initiated causing further increases in the purple azure B-eosin Y complex precipitation and, hence, stain intensity [16]. Indeed, our next experiment confirmed the key role of the Romanowsky-Giemsa effect in the observed pattern of the comet staining. In particular, we stained the comet slides with either azure B alone or Giemsa (Figure 4). It is evident from Figure 4 that azure B alone used at the same concentration it is present in Giemsa stain did not produce distinctly stained and well defined comets (Figure 4a), indicating the requirement of the eosin Y and the Romanoswsky-Giemsa effect for the staining pattern seen in Figure 4b.

## 3. Experimental Section

### 3.1. Isolation of Blood Lymphocytes and Immobilizing Cells into Agarose

Peripheral blood from three healthy 21–28 years old females was used for the experiments. Informed consent was obtained from each human subject for the use of blood cells in this study. The blood was withdrawn using Vacuette^®^ K_2_EDTA blood collection tubes (Greiner Bio-One, Frickenhausen, Germany), followed by centrifugation in a ficoll-verografin density gradient (Histopaque by Sigma-Aldrich, Poole, UK) according to the manufacturer’s recommendations. Lymphocytes were then rinsed and resuspended in phosphate buffered saline (PBS) at a concentration of 10^6^ cells/mL. Two hundred μL of cell suspension was mixed with 600 μL of 1% low melting point agarose (Thermo Scientific, Rockford, IL, USA) in PBS at pH 7.4 and 37.5 °C. Then 75 μL of the lymphocyte-agarose mix was dispensed onto microscope slides pre-coated with 1% normal melting point agarose (Thermo Scientific, Rockford, IL, USA). Following mounting with coverslips, the slides were chilled at 4 °C for 10 min to allow the solidification of the lymphocyte-agarose layer. Slides were then removed and agarose-embedded cells were exposed to X-rays.

### 3.2. Cell Irradiation

The cells were irradiated with either 0 (sham-irradiation), 1, 2, 3, 4, 5 or 6 Gy of X-rays using an X-ray machine RUB RUST-M1 (JSC “Ruselectronics”, Moscow, Russia) at a dose rate of 0.85 Gy/min. X-rays were generated using a 1.5 mm aluminum filter at 200 kV and 2–5 mA. The slides with agarose-embedded cells were kept at 4 °C during irradiations using thermo granules Lab Armor Beads (Thermo Scientific, Rockford, IL, USA).

### 3.3. Comet Assay

The alkaline version of the comet assay was used [10,11]. Briefly, cells were lysed immediately after irradiation in the lysis buffer (2.5 M NaCl, 100 mM Na_2_EDTA, 20 mM Tris-HCl, pH 10.0, 1% Triton X-100 and 10% DMSO) at 4 °C for 1 h protected from light. The agarose embedded cells were then subjected to alkaline denaturation of DNA (300 mM NaOH, 1 mM Na_2_EDTA, pH > 13) at 4 °C for 20 min, followed by electrophoresis at 0.75 V/cm for 20 min at room temperature. The neutralization step, to reconstitute native DNA conformation, was performed by rinsing the slides 3 times in 0.4 M TrisHCl buffer, pH 7.4. The slides were then dried and fixed in 70% ethanol for 10 min.

### 3.4. SybrGreen I Staining of DNA

The comets were stained with SybrGreen I Nucleic Acid Gel Stain (Invitrogen, Eugene, OR, USA) diluted in PBS according to the manufacturer’s instructions.

### 3.5. Giemsa Staining of DNA

The slides were stained for 30 min with a working solution of Giemsa stain prepared from a commercially available stock solution (AppliChem, GmbH, Darmstadt, Germany) according to recommendations of the manufacturer. The slides were then washed 2 × 1 min in Sörensen phosphate buffer (pH 6.8) and air dried.

### 3.6. Visualization of DNA Comets

DNA comets were visualized using a fluorescent microscope Axioscop-40 FL (Carl Zeiss, Jena, Germany) and images were captured using a CCD camera MRc 5 (Carl Zeiss, Jena, Germany) and AxioVision 4.8 software package (Carl Zeiss, Jena, Germany). Images of DNA comets on the captured microphotographs were analyzed using CASP 1.2.2 software (CASPlab, casplab.com, Wroclaw, Poland). Percent DNA in tail was quantified as a measure of DNA damage. One hundred comets were analysed from each of three slides per treatment per donor.

### 3.7. Statistical Analysis

Statistical analysis was carried out using Statistica 8.0 (Statsoft, Tulsa, OK, USA). Regression analysis was done on datasets from each individual donor. The slope coefficients of dose response curves obtained using Giemsa and SybrGreen I stains were compared using the Z-test. Results are presented as mean values ± standard errors of three measurements (three slides per treatment).

## 4. Conclusions

Results presented demonstrate for the first time that Giemsa staining is a suitable staining method for the comet assay. Imaging and quantification of Giemsa-stained DNA comets required only a conventional bright-field microscope equipped with a regular CCD camera and freely available software. Unlike silver staining, another staining method suitable for bright-field microscopy, which is very sensitive to experimental conditions and may produce high non-specific signal, Giemsa staining is very simple and more specific to DNA. The sensitivity of Giemsa staining for the detection of DNA damage in human peripheral blood lymphocytes exposed to various doses of X-rays was similar to the sensitivity normally achieved using fluorescent SybrGreen I staining. This was evident by comparing the radiation dose responses (fitted by linear regressions) generated by the two staining protocols. The involvement of the Romanowsky-Giemsa effect in the unexpectedly high sensitivity of Giemsa staining of DNA comets was confirmed in the experiments showing poor visualization of the comets stained with azure B alone. Other factors contributing to the observed effectiveness of Giemsa stain in the comet assay may be the photostability of the stained DNA and the higher resolution of bright-field imaging compared to fluorescence imaging. Additionally, the stability of Giemsa staining provides an obvious convenience of long-term storage, making possible archiving entire studies for future re-examinations.

Finally, our results open wide opportunities for implementing this inexpensive version of the comet assay for conventional bright-field microscopy in any low-budget research or genotoxicity testing laboratory. It is hoped that the simplicity and affordability of Giemsa stain for the comet assay, at no significant cost to sensitivity, will facilitate new research in biomedicine.

## Figures and Tables

**Table 1.** Results of the regression analysis and the Z-test comparison between slope coefficients for the two staining protocols (*a*, intercept ± SE; *b*, slope coefficient ± SE; *p*, significance level value; *R*^2^, coefficient of determination; *z*, z-score; *p*_z_, significance level value of the Z-test).

Blood donor	Staining	*a*	*p*	*b*	*p*	*R*^2^	*z*	*p*_z_
Donor #1	Giemsa	8.60 ± 1.49	0.002	6.15 ± 0.41	0.000	0.978	-	-
	SybrGreen I	13.70 ± 1.90	0.001	7.62 ± 0.53	0.000	0.977	2.19	0.029
Donor #2	Giemsa	0.80 ± 0.99	0.457	5.56 ± 0.28	0.000	0.988	-	-
	SybrGreen I	6.56 ± 0.98	0.001	6.86 ± 0.27	0.000	0.992	3.34	0.001
Donor #3	Giemsa	5.51 ± 0.43	0.000	5.66 ± 0.12	0.000	0.998	-	-
	SybrGreen I	11.94 ± 0.92	0.000	6.86 ± 0.26	0.000	0.993	4.19	0.000
